# P-555. Use of Long-Acting Cabotegravir/Rilpivirine in People Living with HIV with Renal Transplantation

**DOI:** 10.1093/ofid/ofae631.754

**Published:** 2025-01-29

**Authors:** George Bchech, Cassandra Oehler, Qing Ma, Jennifer Carpenter, Kalathil Sureshkumar, Ryan Rothman, Debra Tives, Chiu-bin hsiao

**Affiliations:** Allegheny General Hospital/Allegheny Health Network, PITTSBURGH, Pennsylvania; Allegheny General Hospital, Positive Health Clinic, Center for Inclusion Health, AHN, Drexel University College of Medicine, Pittsburgh, Pennsylvania; University at Buffalo, Buffalo, New York; Allegheny General Hospital/Allegheny Health Network, PITTSBURGH, Pennsylvania; Allegheny General Hospital/Allegheny Health Network, PITTSBURGH, Pennsylvania; Allegheny General Hospital/Allegheny Health Network, PITTSBURGH, Pennsylvania; Allegheny Singer Research Institute/Allegheny Health Network, Pittsburgh, Pennsylvania; Allegheny General Hospital, Positive Health clinic, Center for Inclusion Health, AHN; Drexel University, College of Medicine, Pittsburgh, Pennsylvania

## Abstract

**Background:**

Cabotegravir/Rilpivirine (CAB/RPV) represents a novel combination of long-acting injectable antiretroviral therapy (LAIART). Although the unique benefits of LAIART have been demonstrated in large clinical trials among people with HIV, including a favorable safety profile, few drug-drug interactions, and viral control without a daily oral pill requirement, evidence of LAIART use among PWH diagnosed with end-stage chronic kidney disease (CKD) and during kidney transplantation (KT) remains sparse.

**Figure:**
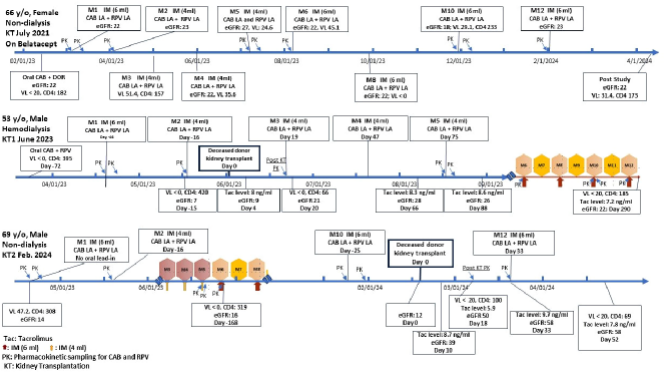

Detailed timeline of all 3 patients in the study.

**Methods:**

A phase IV clinical trial CAPRI (NCT05601128) was designed to evaluate the pharmacokinetics and safety of long-acting CAB/RPV among PWH with CKD >= stage 4 or ongoing hemodialysis. Three enrolled participants were included, in this analysis because of their KT status, of them, one KT before the trial and two KT during the trial. The peri- and post-operative effects of CAB/RPV LA were evaluated focusing on viral load, CD4+ counts, and potential drug interactions. According to protocol, CAB/RPV PK samples were obtained and planned to be measured and analyzed.

**Results:**

All three participants have been virally suppressed (< 50 copies/ml) by CAB/RPV LA with stable CD4+ counts while maintaining appropriate graft function during 12months of study period. The timeline for each participant was depicted in the **Figure** and details were summarized in terms of virologic and immunologic outcomes as well as renal function at different study periods. CAB/RPV LA was well tolerated, without major related adverse events noted. The renal function was significantly improved, dialysis free, in two participants receiving KT during the trial whereas it remained stable, dialysis free, in the participant with prior KT. The reported tacrolimus trough concentrations were maintained within the therapeutic range to protect the graft function.

**Conclusion:**

The safety and efficacy of CAB/RPV LA have been observed in these participants, supporting its potential use among those with severe renal impairment and kidney transplantation. Additional advantages including lack of adherence barriers and low drug interaction potential are likely to increase viral suppression and improve overall quality of life in this special population.

**Disclosures:**

**Chiu-bin hsiao, MD**, Viiv, Gilead: Grant/Research Support

